# The Effects of Remifentanil, Dexmedetomidine, and Metoral as Adjuncts to Thiopental on Hemodynamic Status After Electroconvulsive Therapy in Patients with Major Depressive Disorder: A Randomized Controlled Clinical Trial

**DOI:** 10.5812/aapm-139383

**Published:** 2023-09-19

**Authors:** Nastaran Tajabadi, Alireza Kamali, Anita Alaghmand, Hamidreza Jamilian, Shirin Pazooki, Amin Tajerian

**Affiliations:** 1 Department of Student Research Committee, Arak University of Medical Sciences, Arak, Iran; 2 Department of Anesthesiology and Critical Care, Arak University of Medical Sciences, Arak, Iran; 3 Department of Psychiatry, School of Medicine, Arak University of Medical Sciences, Arak, Iran; 4 School of Medicine, Arak University of Medical Sciences, Arak, Iran

**Keywords:** Remifentanil, Dexmedetomidine, Metoral, Thiopental, Major Depressive Disorder, Electroconvulsive Therapy

## Abstract

**Background:**

Depression is a prevalent mental disorder affecting more than 300 million people of all ages globally. Despite being the first-line treatment for depression, antidepressant medications are only effective for 60% - 70% of patients. Electroconvulsive therapy (ECT) is an effective treatment for severe cases, although it can result in short-term side effects.

**Objectives:**

This study aimed to compare the effectiveness of remifentanil, dexmedetomidine, and metoral as premedications for ECT in patients with major depressive disorder (MDD).

**Methods:**

In this prospective double-blinded randomized controlled clinical trial, a total of 120 MDD patients aged 18 - 60 were included. They were randomly assigned to receive remifentanil, dexmedetomidine, or metoral in combination with thiopental before ECT. Hemodynamic responses (mean arterial blood pressure, pulse rate, arterial blood oxygen saturation), seizure duration, recovery time, agitation scores, and patient satisfaction scores (reverse coded) were measured and compared.

**Results:**

Dexmedetomidine exhibited superior hemodynamic control with lower mean arterial blood pressure (P < 0.001) and pulse rate (P < 0.001) than remifentanil and metoral. Patients receiving dexmedetomidine or remifentanil showed reduced agitation (P < 0.001) and better satisfaction than the metoral group (P < 0.001). Remifentanil displayed intermediate outcomes, while metoral exhibited the least favorable results. Seizure duration was not significantly different between the dexmedetomidine and remifentanil groups (P = 0.843).

**Conclusions:**

Dexmedetomidine is considered the most satisfactory group due to the better control of blood pressure, heart rate, and agitation and better patient satisfaction despite the longer recovery time.

## 1. Background

Depression stands as a prevalent mental disorder, impacting over 300 million individuals worldwide across all age groups, with a global point prevalence of 4.7% (1, 2). In addition to its extraordinarily high prevalence, it also grows over time (3). From 2005 to 2018, there was a noticeable rise in the number of adults diagnosed with major depressive disorder (MDD) in the USA, escalating from 13.7 million to 17.5 million. Additionally, the prevalence rate experienced a modest increase during this period, climbing from 6.8% to 7.1% (4, 5).

Antidepressant medications are only effective for 60% - 70% of patients, and the response rate to the initial prescribed agent can be as low as 50% (6). Among individuals who do not respond to antidepressant treatment, approximately 10% - 30% experience treatment-resistant symptoms, which may manifest as difficulties in social and occupational functioning, declined physical health, suicidal ideation, and heightened healthcare utilization (7). According to findings from the sequenced treatment alternatives to relieve depression (STAR*D) trial, approximately 50% - 66% of patients with depression do not achieve full recovery while on antidepressant medications. Furthermore, only one-third of patients experience a remission of their depressive symptoms (8).

Electroconvulsive therapy (ECT) emerges as the optimal treatment option for severe cases of treatment-resistant MDD, where a swift response is essential (9). Electroconvulsive therapy has demonstrated a rapid and substantial improvement in depressive symptoms, leading to remission in a significant proportion of treated patients. According to the Consortium for Research in ECT (CORE) findings, 75% of patients completing a short course of ECT during an acute episode of depression achieved remission (10). Furthermore, the CORE group reported that patients with the psychotic subtype of depression displayed higher response rates to ECT than those without psychosis (11). The guidelines provided by the American Psychiatric Association (APA) Task Force on ECT recommend considering ECT primarily for MDD patients who show inadequate response to or intolerance of antidepressant medications, have previously responded positively to ECT, and require a rapid and definitive response (6).

Electroconvulsive therapy is considered a low-risk treatment with no absolute contraindications. Nevertheless, patients and doctors should be mindful of potential side effects, including cognitive disorders that typically resolve quickly, rare seizures, and short-term effects like headache, nausea, vomiting, and fatigue (6). In addition, ECT typically induces a temporary rise in blood pressure and tachycardia, which may result in a pronounced hyperdynamic response in the patient (12). Another short-term effect is postictal agitation, characterized by restlessness and confusion in the patient (13, 14). General anesthesia and neuromuscular blockers are administered during ECT to ensure patient comfort and safety. The use of induction drugs for general anesthesia creates a more stable cardiovascular state, facilitating the occurrence of more prolonged seizures (15).

Numerous studies have examined the impact of anesthesia drugs on various outcomes following ECT. These outcomes include the rate of recovery from disorientation (16), levels of agitation (17, 18), patient satisfaction (19-21), hemodynamic responses (15, 22), seizure duration (22, 23), and the time required for recovery (12). Moreover, anesthesia drugs, such as dexmedetomidine, propofol, thiopental, ketamine, remifentanil, methohexital, and alfentanil, have been thoroughly evaluated for their effectiveness in providing anesthesia before ECT (15-23).

However, we found no study directly comparing the effects of remifentanil, dexmedetomidine, or metoral in combination with an anesthetic inducer for anesthesia before ECT. Remifentanil, known for its short-acting opioid properties, demonstrates potent analgesic effects and effectively reduces blood pressure and heart rate when used as an adjuvant in anesthesia (12). Dexmedetomidine, an α2-adrenoceptor agonist, possesses sedative and analgesic properties contributing to hemodynamic stability during procedures (15). On the other hand, metoral acts as a selective β1 receptor blocker, leading to reductions in cardiac output, heart rate, and blood pressure (24). Despite their characteristics, a direct comparison of these agents' effects during anesthesia for ECT is yet to be undertaken.

## 2. Objectives

Our objective was to assess and compare the effects of introducing remifentanil, dexmedetomidine, or metoral alongside thiopental on various parameters, including hemodynamic responses, agitation levels, patient satisfaction, seizure activity duration, and recovery time in individuals with MDD undergoing ECT.

## 3. Methods

### 3.1. Patients and Design

This prospective double-blinded randomized controlled clinical trial was registered and approved by the Iranian Registry of Clinical Trials (IRCT) (registration number: IRCT20141209020258N178). It also received approval from the Institutional Ethics Committee of Arak University of Medical Sciences on October 9, 2022. The study was conducted at Amirkabir Hospital, Arak, Iran, from October 2022 to March 2023. Prior to enrollment, patients or their legal guardians provided written informed consent. The study adhered to the Consolidated Standards of Reporting Trials (CONSORT) guidelines (please refer to the supplementary materials for further details).

Patients aged 18 - 60, of both genders, who were diagnosed with MDD and deemed suitable candidates for ECT based on a joint evaluation by a psychiatrist and an anaesthesiologist, were included in this study. Exclusion criteria encompassed patients with American Society of Anesthesiologists (ASA) physical status III or higher, recent pregnancy, organ failure, alcohol or substance abuse, low pseudocholinesterase levels, glaucoma, premedication with drugs like β-adrenergic blockers, and known or family history of adverse reactions to dexmedetomidine, remifentanil, metoral, or thiopental, as well as any contraindications for ECT, such as brain space-occupying lesions, increased intracranial pressure, and recent myocardial infarction.

**Figure 1.**
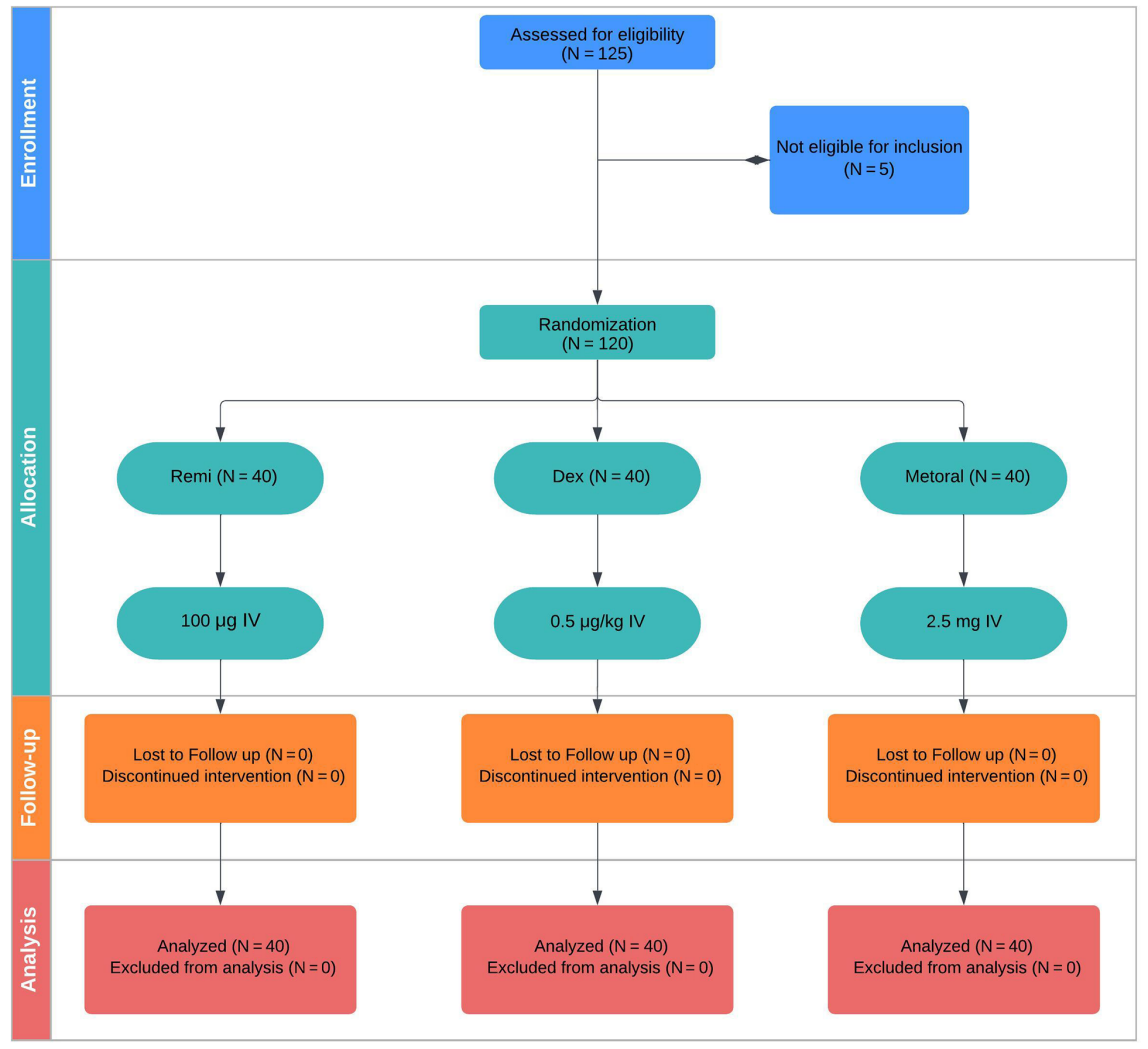
The consolidated standards of reporting trials diagram shows the flow of the clinical randomized trial. Note: Dex, dexmedetomidine; Remi, remifentanil.

Patients were randomly assigned to receive remifentanil, dexmedetomidine, or metoral, utilizing computer-generated permuted blocks in a 1: 1: 1 ratio. Each block consisted of 12 participants, and the researchers, clinicians (excluding the chief anesthesiologist), patients, and families were kept unaware of their group assignments (Figure 1). 

The trial's design was formulated by the authors, who were also responsible for data collection and analysis, ensuring accuracy, completeness, and adherence to the protocol. Eligible subjects for ECT, as diagnosed by psychiatrists (A. A. and H. J.), also underwent an anesthesiologist (A. K.) assessment based on the study protocol to determine their inclusion in the study.

### 3.2. Trial Interventions

Remifentanil, dexmedetomidine, and metoral were prepared in identical intravenous fluid bags, which were covered with opaque plastic to maintain the study’s masking. Nurse anesthetists ensured that the intravenous tubing was also covered with opaque materials, containing the same volume of 5cc, and confirmed the proper placement of the covers before any study researchers or clinicians entered the ECT room.

In group A, remifentanil was administered at a dosage of 100 μg, group B received 0.5 μg/kg of dexmedetomidine, and group C received an intravenous infusion of metoral at 2.5 mg. Following premedication, all patients underwent anesthesia induction with sodium thiopental (3 mg/kg). During the procedure, skeletal muscle relaxation was achieved using succinylcholine, which was administered intravenously (IV) at a dose of 0.5 mg/kg for all patients. Additionally, atropine was injected as a prophylactic measure against bradycardia after using succinylcholine.

Hyperventilation was performed on all patients using 100% oxygen delivered through a bag-valve mask. Once complete anesthesia was achieved, bilateral electrical stimulation was applied based on the psychiatrist's opinion, using electrodes on both sides of the temporal region. Electric shock was applied with the desired energy level specified by the psychiatrist, after which patients were reoxygenated and transferred to the recovery room.

### 3.3. Measurements

After anesthesia induction and immediately following the ECT procedure, each patient's mean arterial blood pressure, pulse rate, and arterial blood oxygen saturation levels were recorded.

Recovery time was precisely defined and recorded as the time interval (in minutes) from the administration of succinylcholine until the subjects responded to verbal commands and were able to open their eyes. Additionally, seizure duration was recorded in seconds. In order to measure patients' satisfaction, a Likert scale satisfaction survey was used. Patients scored their levels of satisfaction as 1 = feeling happy and calm, 2 = experiencing no discomfort or dissatisfaction, 3 = having moderate satisfaction, and 4 = unsatisfied and unwilling to undergo the same technique (25). Post-recovery agitation levels were recorded using a 5-point Likert agitation score, including 1 = sleeping, 2 = being awake and calm, 3 = being irritable and crying, 4 = experiencing inconsolable crying, and 5 = displaying severe agitation or willingness to wake up from bed or sit on the bed and shriek (26).

Data were recorded by N. T., who was kept unaware of the grouping information to ensure the study's masking.

### 3.4. Statistical Analysis

Descriptive statistics were employed to summarize the data characteristics, with categorical variables presented as frequencies and percentages and continuous variables presented as mean ± standard deviation. The *t*-tests, analysis of variance (ANOVA), chi‑square, and repeated measurement tests were utilized for inferential analyses. After conducting an ANOVA and finding significant differences among the groups, we further explored pairwise comparisons using Tukey's honestly significant difference (HSD) test. Statistical significance was set at a P-value of less than 0.05.

The statistical analysis was performed using IBM SPSS Statistics 27 (IBM Corp., USA). Additionally, Plotly, an open-source library for Python, was utilized to create data visualizations.

## 4. Results

Finally, 120 patients were enrolled in this trial (mean age = 37.20 ± 7.08). In this study, we included 84 (70%) males (mean age = 37.23 ± 6.62) and 36 (30%) females (mean age = 37.14 ± 8.15). There was no significant difference in age between male and female patients (P = 0.955); see Table 1. 

**Table 1.** Demographics ^a^

Demographics		N = 40			Test (P-Value)	
Remi	Dex	Met	ANOVA	Chi2
**Mean age**	37.25 ± 6.935	37.20 ± 8.049	37.15 ± 6.343	0.998	
**Gender**					0.888
Male	28 (70)	29 (72.5)	27 (67.5)		
Female	12 (30)	11 (27.5)	13 (32.5)		

Abbreviations: Dex, dexmedetomidine; Remi, remifentanil; Met, metoral; ANOVA, analysis of variance; Chi, chi-square test.

^a^ Data are expressed as mean ± standard deviation and frequency (%).

**Figure 2.**
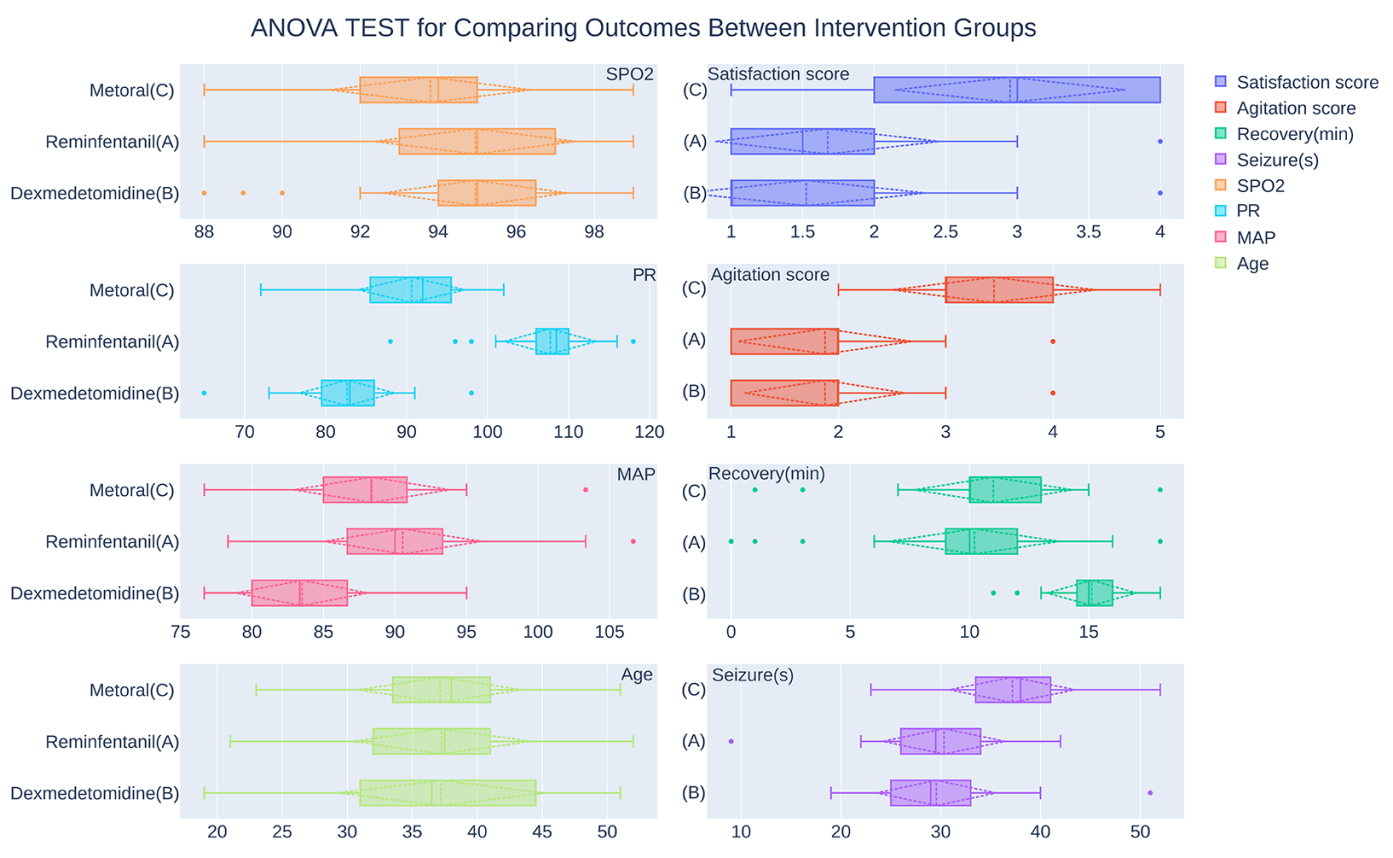
The analysis of variance test to compare outcomes between intervention groups. Dashed lines indicate the mean and ± one standard deviation interval from the mean. Dots indicate outliers.

As shown in Figure 2 and Table 2, patients had mean arterial pressure scores of 90.53 ± 5.56 in the remifentanil group (A); 83.49 ± 4.66 in the dexmedetomidine group (B); and 88.37 ± 5.51 in the metoral group (C). A one-way ANOVA revealed a statistically significant difference in the mean arterial pressure score between at least two intervention groups (P < 0.001). Post-hoc analyses employing the Tukey HSD test revealed that the mean arterial pressure between the metoral and remifentanil groups was not significantly different (P = 0.161). However, the mean arterial pressure score in group B was significantly lower than in both groups A (P < 0.001) and C (P < 0.001); see Table 3. 

**Table 2.** The Effects of Remifentanil, Dexmedetomidine, and Metoral as Adjuncts to Thiopental on Hemodynamic Status, Anesthetic Effects, and Agitation and Satisfaction Scores ^a^

Variables	N = 40			P-Value
	Remi	Dex	Met	ANOVA
**MAP**	90.53 ± 5.56	83.49 ± 4.66	88.37 ± 5.51	< 0.001
**PR**	107.75 ± 5.71	82.65 ± 5.93	90.63 ± 6.67	< 0.001
**SPO** ^**2**^	94.95 ± 2.62	94.95 ± 2.39	9380 ± 2.61	0.070
**Recovery duration (min)**	10.20 ± 3.61	15.13 ± 1.89	11.00 ± 3.351	< 0.001
**Seizure duration (sec)**	30.33 ± 6.20	29.55 ± 6.04	37.17 ± 6.40	< 0.001
**Agitation score**	1.88 ± 0.82	1.88 ± 0.75	3.45 ± 0.95	< 0.001
**Satisfaction score**	1.68 ± 0.79	1.53 ± 0.84	2.95 ± 0.81	< 0.001

Abbreviations: Dex, dexmedetomidine; Remi, remifentanil; Met, metoral; MAP, mean arterial pressure; PR, pulse rate; SPO, blood oxygen saturation.

^a^ Data are expressed as mean ± standard deviation and analyzed by the analysis of variance.

**Table 3.** Tukey’s Honestly Significant Difference Post Hoc Analysis ^a^

Variables ^b^	Groups			P-Value
	Remi	Dex	Met	
**MAP**				
Subset 1		83.49 ± 4.66		1.0
Subset 2	90.53 ± 5.56		88.37 ± 5.51	0.161
**PR**				
Subset 1		82.65 ± 5.93		1.0
Subset 2			90.63 ± 6.67	1.0
Subset 3	107.75 ± 5.71			1.0
**Seizure duration**			107.75 ± 5.71	1.0
Subset 1	30.33 ± 6.20	29.55 ± 6.04		0.843
Subset 2			37.17 ± 6.40	1.0
**Recovery duration**				
Subset 1	10.20 ± 3.61		11.00 ± 3.351	0.471
Subset 2		15.13 ± 1.89		1.0
**Agitation score**				
Subset 1	1.88 ± 0.82	1.88 ± 0.75		1.0
Subset 2			3.45 ± 0.95	1.0
**Satisfaction score**				
Subset 1	1.68 ± 0.79	1.53 ± 0.84		0.693
Subset 2			2.95 ± 0.81	1.0

Abbreviations: Dex, dexmedetomidine; Remi, remifentanil; Met, metoral; MAP, mean arterial pressure; PR, pulse rate.

^a^ data are expressed as mean ± standard deviation and analyzed by Tukey’s honestly significant difference post hoc analysis

^b^ Subset for alpha = 0.05.

A statistically significant difference was seen in the mean pulse rate values between groups A (107.75 ± 5.71), B (82.65 ± 5.93), and C (90.63 ± 6.67) using one-way ANOVA test (P < 0.001). Tukey's HSD test showed that the pulse rate in the remifentanil group was significantly higher than in groups B (P < 0.001) and C (P < 0.001). Additionally, the mean pulse rate in the metoral group was higher than in the dexmedetomidine group (P < 0.001); see Table 3. 

The mean blood oxygen saturation values for groups A (94.95 ± 2.62), B (94.95 ± 2.39), and C (93.8 ± 2.61) exhibited no statistically significant difference, with a P-value of 0.070; see Table 2. 

As demonstrated in Table 2, the seizure duration was compared between intervention groups, and there was a statistically significant difference (P < 0.001) between groups A (30.33 ± 6.20), B (29.55 ± 6.04), and C (37.17 ± 6.40) seizure times. Tukey’s post hoc test showed that the mean seizure time was significantly higher in group C than in group A (P < 0.001) or B (P < 0.001). However, there was no significant difference between the mean seizure times of groups A and B (P = 0.843); see Table 3. 

The recovery time had a statistically significant difference (P < 0.001) between groups A (10.20 ± 3.61), B (15.13 ± 1.89), and C (11.00 ± 3.35). The post hoc test showed that the mean recovery time was significantly higher in group B than in group A (P < 0.001) or C (P < 0.001), but there was no significant difference between groups A and C recovery time (P = 0.471); see Table 3. 

As Table 2 shows regarding the patients' mean agitation and satisfaction rates after ECT, there was a significant difference (both P < 0.001) in agitation and satisfaction scores between the intervention groups. Tukey’s post hoc test showed that patients taking metoral in group C had significantly higher agitation (P < 0.001) and satisfaction scores (P < 0.001) than groups A and B. However, there was no significant difference between groups A and B regarding agitation (P = 1.0) and satisfaction rates (P = 0.693); see Table 3. 

## 5. Discussion

Anesthesia and neuromuscular blockade are necessary to prevent mental and physical damage during ECT. In order to find a better combination with thiopental that could help minimize acute hemodynamic alterations, reduce cardiovascular side effects, and provide a better experience for patients, we compared the effects of adding remifentanil, dexmedetomidine, and metoral to the regimen.

We found that the mean arterial pressure score was significantly lower with dexmedetomidine than in other groups. Comparing remifentanil and metoral, we found no significant difference in the mean arterial pressure. We also found that the mean pulse rate was significantly lower with dexmedetomidine premedication than the other two groups. Comparing remifentanil and metoral, we found that patients receiving metoral premedication in combination with thiopental had significantly lower pulse rates than the remifentanil group. The intervention groups showed no statistically significant difference in the arterial blood oxygen saturation levels.

Since dexmedetomidine premedication, compared to remifentanil or metoral, can lower mean arterial pressure and heart rate more effectively, and there is no significant difference in arterial blood oxygen saturation, we can conclude that administering dexmedetomidine in combination with thiopental for ECT anesthesia is a better choice for improving the patients' hemodynamic status.

When comparing seizure durations, a noteworthy difference emerged between the intervention groups. Specifically, the metoral group exhibited a significantly longer seizure duration than the other two groups. However, no significant difference was observed between the dexmedetomidine and remifentanil groups.

Moreover, a comparison of the mean duration of recovery time among the intervention groups revealed a significant difference. The dexmedetomidine group had a significantly longer mean recovery duration than the other two groups. However, there was no significant difference between the remifentanil and metoral groups in terms of recovery time.

According to our findings, the satisfaction and agitation scores in the metoral group were significantly higher than those in the other two groups. However, there was no statistically significant difference in the satisfaction and agitation scores between the remifentanil and dexmedetomidine groups.

Our findings were in line with the results of Heidarbeigi et al.’s study investigating the impact of adding dexmedetomidine or remifentanil to thiopental in patients with mood disorders undergoing ECT (27). Their study revealed that the dexmedetomidine group had lower mean blood pressure and heart rate than the remifentanil group. Although the seizure duration was longer in the remifentanil group than in the dexmedetomidine group, this difference was not statistically significant. However, the recovery time was significantly longer in the dexmedetomidine group than in the remifentanil group. Additionally, no significant differences were observed between the two groups regarding blood oxygen saturation agitation, and satisfaction scores.

Furthermore, Mohammadi et al.’s study in 2020 investigating the impact of adding dexmedetomidine and metoral to thiopental in patients with mood disorders undergoing ECT revealed that the dexmedetomidine group had lower mean arterial pressure and pulse rate than the metoral group. However, no significant difference was observed between the two groups in arterial blood oxygen saturation. Moreover, the recovery time was longer in the dexmedetomidine group, while the metoral group exhibited higher agitation scores than the dexmedetomidine group. Additionally, the satisfaction score was higher in the dexmedetomidine group. These results were consistent with the findings of our study (28).

In their study in 2009 investigating the impact of dexmedetomidine and midazolam treatment on reducing agitation during the ECT procedure, Mizrak et al. found that the group receiving dexmedetomidine had longer seizure durations than the other groups. These results contrasted our findings observing a shorter seizure duration in the dexmedetomidine group. The differences in the drug combinations used in the two studies appear to have contributed to the disparate outcomes (17).

Aksay et al.’s study in 2017 found that dexmedetomidine could effectively manage postictal agitation following ECT, aligning with the findings of our study (13).

Finally, the comprehensive analysis of the results reveals that the combination of dexmedetomidine and thiopental exhibits better hemodynamic control, as indicated by lower mean arterial pressure and heart rate, along with comparable arterial blood oxygen saturation levels. Moreover, patients receiving dexmedetomidine and thiopental demonstrated lower agitation and higher satisfaction scores than the other two groups. These findings suggest that dexmedetomidine contributes to improved hemodynamic stability and subsequently leads to higher patient satisfaction following ECT.

In this study, dexmedetomidine was identified as the most favorable adjuvant, being recommended as the top choice. The combination of remifentanil and thiopental was ranked as the second-best option. This ranking was supported by the observation that patients receiving remifentanil display lower satisfaction and agitation scores than those receiving the combination of metoral and thiopental.

### 5.1. Conclusions

Dexmedetomidine is considered the most satisfactory group due to the better control of blood pressure, heart rate, and agitation and better patient satisfaction despite the longer recovery time. After that, remifentanil can be a suitable option for use during anesthesia induction for electroshock therapy due to proper blood pressure control and patient satisfaction.

## Data Availability

The dataset presented in the study is available on request from the corresponding author during submission or after publication.

## Clinical Trial Registration Code:

IRCT20141209020258N178.
